# The debate between electricity and heat, efficacy and safety of irreversible electroporation and radiofrequency ablation in the treatment of liver cancer: A meta-analysis

**DOI:** 10.1515/biol-2022-0991

**Published:** 2024-12-18

**Authors:** Rong Xing, Yutong Liu, Yang Liu, Haihong Jiang, Chao Liu, Jiru Du

**Affiliations:** School of Medical Devices, Shanghai University of Medicine & Health Sciences, Shanghai, 201318, China; Liver Disease Center, The Affiliated Hospital of Qingdao University, Shanghai, 266003, China; School infirmary, Fudan University, Shanghai, 200433, China

**Keywords:** irreversible electroporation, radiofrequency, liver cancer, meta-analysis

## Abstract

Both irreversible electroporation (IRE) and radiofrequency ablation (RFA) are viable ablation methods for localized treatment of liver tumors. We conducted a meta-analysis to access the efficacy and safety of IRE and RFA in liver cancer treatment. Clinical studies on IRE and RFA for the treatment of liver cancer were collected from PubMed and CNKI until June 2023. We screened the literature for ablation success rates at 1 month post-operation, extracting keywords such as “ablation success rate,” “technical success rate,” “recurrence rate,” and “complication” for meta-analysis. A total of 37 articles were included: 24 related to RFA involving 1,685 cases and 13 related to IRE involving 524 cases. The results demonstrate that ablation success rates at post-operative 1 month for IRE and RFA were 86% (95% CI: 82–89%) and 87% (95% CI: 81–92%), respectively. Technical success rates were 96% (95% CI: 88–100%) and 99% (95% CI: 96–100%). In addition, the recurrence rate was 16% (95% CI: 12–22%) in RFA group and 16% (95% CI: 9–23%) in IRE group. In terms of safety, the RFA had a complication rate of 28% (95% CI: 10–50%) and the IRE had a rate of 26% (95% CI: 13–43%). In conclusion, IRE and RFA exhibit similar ablation success rates at 1 month post-operation and comparable complication rates, making them both safe and effective treatment options.

## 1 Introduction

Liver cancer is a prevalent malignant tumor of the digestive system worldwide. According to the new data released by GLOBOCAN 2020, the global annual incidence of liver cancer has reached 906,000 new cases, with 830,000 deaths, ranking it sixth in incidence and third in mortality among malignant tumors. Both incidence and death are rising [1].

Clinicians typically choose treatment methods based on the tumor’s characteristics, the patient’s liver function, and overall health. If the focus is limited to the liver, surgical treatment (such as partial hepatectomy or liver transplantation) or local ablation is preferred. Systemic therapy is usually the treatment of choice for extrahepatic metastases [2]. Local ablation treatments mainly include radiofrequency ablation (RFA), microwave ablation (MWA), cryoablation, laser ablation, irreversible electroporation (IRE), etc.

RFA is an earlier ablation method used in clinical studies. Its principle is to utilize a high-frequency electric current to excite the ions in the tissue to generate high-frequency oscillation, and the ions rub against each other and collide to generate heat, resulting in coagulation necrosis of tumor cells [3]. IRE is a novel non-thermal tumor ablation technique. Its principle is to generate a high external electric field by electrode needles in the target area, resulting in the formation of nanometer-sized pores in the cell membrane, causing cell apoptosis due to homeostasis imbalance [4]. Compared with RFA, IRE has two potential advantages [1]. It is tissue-selective due to varying electric field ablation thresholds, allowing it to preserve critical structures such as blood vessels, bile ducts, and nerves [2]. It lacks thermal deposition effects, preventing inadequate tissue damage near blood vessels due to uneven heating.

The ablation success rate is a key indicator for evaluating the efficacy of ablation techniques. It is defined as the absence of enhancement in the arterial phase of the ablated tumor lesion on dynamic contrast-enhanced CT or MRI scans, indicating complete tumor necrosis. Observing whether the tumor is completely ablated in the short-term postoperative period is a very important evaluation endpoint. It can not only reflect the short-term efficacy, but also provide an early reference for whether the patients require secondary ablation or other therapies as soon as possible. To date, no meta-analysis has specifically addressed the ablation success rate 1 month after ablation. Therefore, in order to assess the safety and efficacy of these two procedures, we concentrated on gathering data on the ablation success rate and complications about 1 month after RFA and IRE ablation.

## 2 Methods

### 2.1 Search strategy

All clinical studies on IRE and RFA for the treatment of liver cancer were searched from PubMed and CNKI since their database established until June 2023. The search terms were “Radiofrequency AND liver cancer” and “Irreversible Electroporation AND liver cancer,” and the language of the articles was limited to Chinese and English.

### 2.2 Inclusion and exclusion criteria

Inclusion criteria included: (1) patients with primary and metastatic liver cancer, (2) treatment with RFA/IRE technology, (3) clinical research, and (4) evaluation indexes include the success rate of ablation in about 1 month.

Exclusion criteria included: (1) repeated articles; (2) articles with unclear data sources; (3) reviews, conference articles, supporting materials, case reports, and patents; and (4) articles not containing content related to ablation success rate.

### 2.3 Data collection and synthesis

Two researchers independently reviewed the titles and abstracts of each study, as well as the full texts of studies that met the inclusion criteria. A standardized data collection form was used to extract key information, and any disagreements were resolved through discussion. The extracted data include the main author of the literature, publication year, study population characteristics, duration of follow-up, ablation success rate, technical success rate, recurrence rate, and complications.

The R4.1.3 meta-package was used for all of the meta-analyses. The *I* ^2^ statistic can be used to evaluate heterogeneity among research. When *I* ^2^ > 50% (*P* < 0.05), it may be assumed that there is heterogeneity, and the estimates should be summarized using the random effects model; when *I* ^2^ < 50% (*P* > 0.05), it can be assumed that the studies are homogeneous, and the fixed effects model is preferable to the random effects model. In this study, the results of the meta-analysis were presented using forest plots.

### 2.4 Literature quality evaluation

Of the included 37 studies, 10 were randomized controlled trials and 27 were cohort studies. Thus, we use the Cochrane ROB and Newcastle-Ottawa Score (NOS) to assess the quality of the included studies in meta-analyses. NOS was rated more than 6 stars, which is considered as relatively high-quality.

## 3 Results

**Figure 1:**
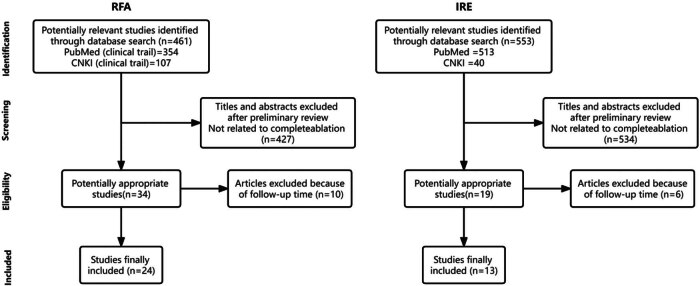
Details of the study selection process.

Through database searches, 461 and 553 published articles were identified by RFA and IRE, respectively, and screened independently by two reviewers. The study flow and reasons for exclusion are detailed in Figure 1. A total of 37 articles were finally included, of which 24 were RFA and 13 were IRE.

### 3.1 Characteristics of included studies in the final analysis

A total of 37 studies were used to extract at least one result, and all of them gave information on the success rates of ablation. In addition, 11 of them provided information on technical success rates, 15 on recurrence rates, and 17 on complications. The characteristics of the included studies are described in Table 1.

**Table 1:** Characteristics of included studies

Studies	Country	No. of patients	Patient age	Data collection time	Ablation rate (%)	Adverse complications (%)
**RFA**						
Liu [5]	China	59	60	2003.09–2012.10	84.75	30.51
Huang et al. [6]	China	146	54.4	2005.03–2009.03	85.62	
Song [7]	China	103	39–81	2010.09–2012.03	72.82	
Fan and Li [8]	China	41	NA	2011.01–2014.01	85.37	
Du et al. [9]	China	59	NA	2004.03–2006.03	81.36	
Wang et al. [10]	China	21	48.3	2010.01–2012.03	90.48	0.00
Song et al. [11]	China	27	Group 1: 60.4	2004.01–2006.04	62.96	
			Group 2: 58.8			
Liu et al. [12]	China	106	Group 1: 54.5	2011.03–2012.03	75.47	
			Group 2: 52.5			
Zhang et al. [13]	China	20	61.4	2011.10–2013.03	80.00	100.00
Zhang et al. [14]	China	57	Group 1: 55.3	2008.12–2010.10	92.98	
			Group 2: 57.8			
Tang et al. [15]	China	48	59.8	2014.01–2015.05	87.50	
El-Kady et al. [16]	Egypt	40	50.6	2008.05–2010.11	85.00	
Azab et al. [17]	Egypt	66	46–77	2005–2008	71.21	
Giorgio et al. [18]	Italy	13	70	2005.01–2008.01	76.92	23.08
Gadaleta et al. [19]	Italy	51	70	2006.07–2007.10	88.24	
Hou et al. [20]	China	177	Group 1: 59.3	2003.01–2007.06	90.40	9.09
			Group 2: 61.3			
Wong et al. [21]	China	208	66.3	2004.01–2006.08	88.94	22.73
Orlacchio et al. [22]	UK	15	71.5	2009–2011	86.67	53.33
Poon et al. [23]	China	80	Group 1: 63.5	2001.05–2002.10	91.25	
			Group 2: 64			
Orlacchio et al. [24]	Italy	8	72.43	2010.12–2013.10	87.50	75.00
Ruzzenente et al. [25]	Italy	104	67.9	1998.01–2003.06	73.08	16.92
Abdelaziz et al. [26]	Egypt	52	56.8	2009.02	94.23	11.11
Schullian et al. [27]	UK	114	65.5	2003–2018	95.61	7.69
Di Costanzo et al. [28]	Italy	70	70	2009.01–2012.09	97.14	
**IRE**						
Sutter et al. [29]	France	75	65.4	2012.03–2015.06	77.33	18.97
Li et al. [30]	China	28	58.7	2019.04–2019.09	96.43	17.39
Freeman et al. [31]	Australian	33	65.2	2008.12–2019.10	87.88	69.57
Cheung et al. [32]	Australian	18	70	2008.11–2009.12	72.22	
Mafeld et al. [33]	USA	59	64	2013–2017	74.58	17.31
Niessen et al. [34]	Germany	103	63.5	2011.10–2015.07	92.23	
Zhou et al. [35]	China	24	21	2016.05–2019.06	79.17	
Beyer et al. [36]	Germany	19	60.3	2014.08–2015.08	94.74	
Fang et al. [37]	UK	69	NA	2014.02–2020.01	81.16	
Eisele et al. [38]	Germany	14	63	—	78.57	
Niessen et al. [39]	Germany	65	59.4	2011.12–2013.06	95.38	27.45
Thamtorawat et al. [40]	Thailad	11	69.2	2014.01–2020.09	100.00	15.38
Cheng et al. [41]	USA	6	61	2011–2013	83.33	

### 3.2 Assessment of effectiveness outcomes

**Figure 2:**
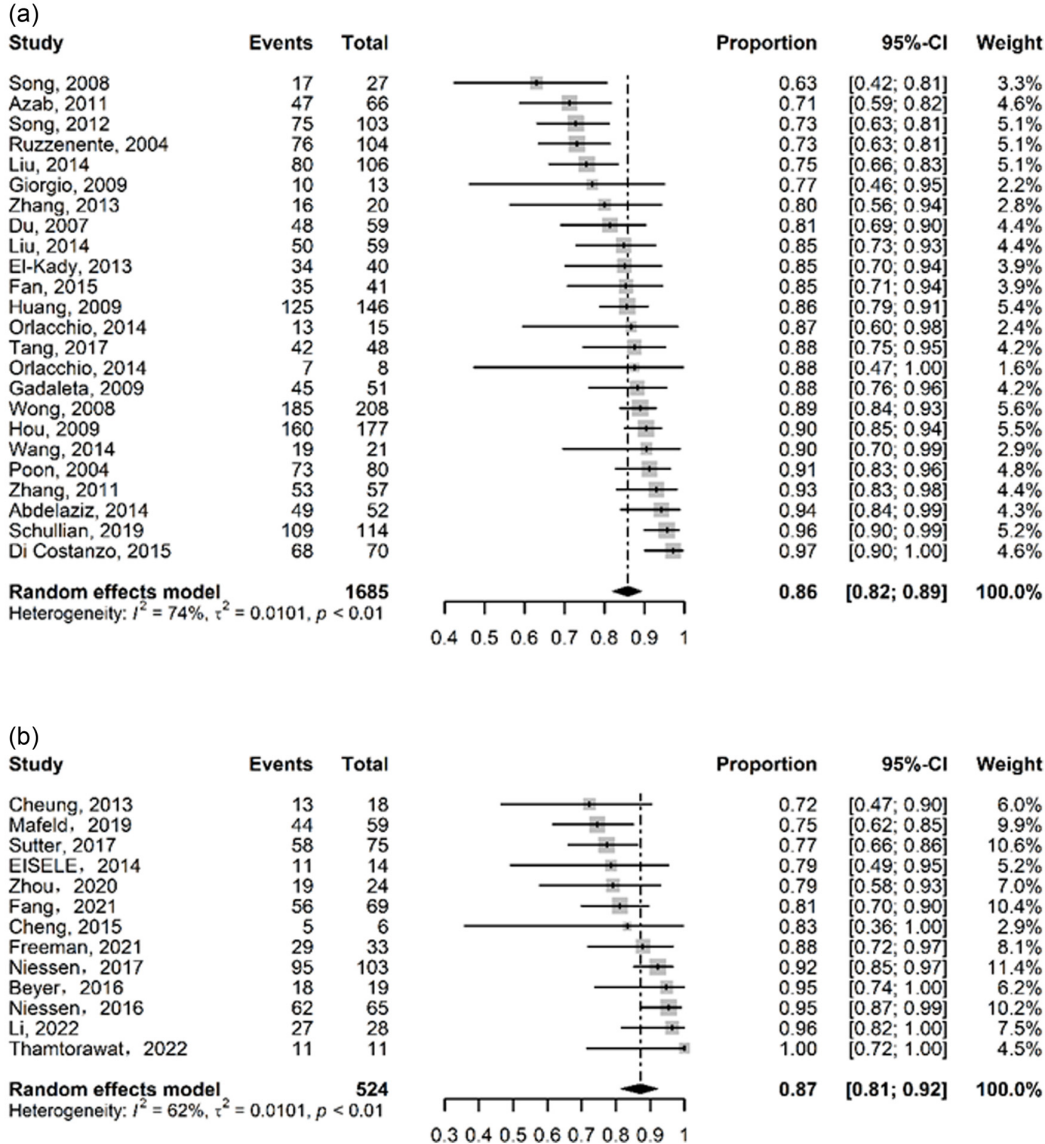
Forest plot for complete ablation rate for (a) RFA and (b) IRE.

**Figure 3:**
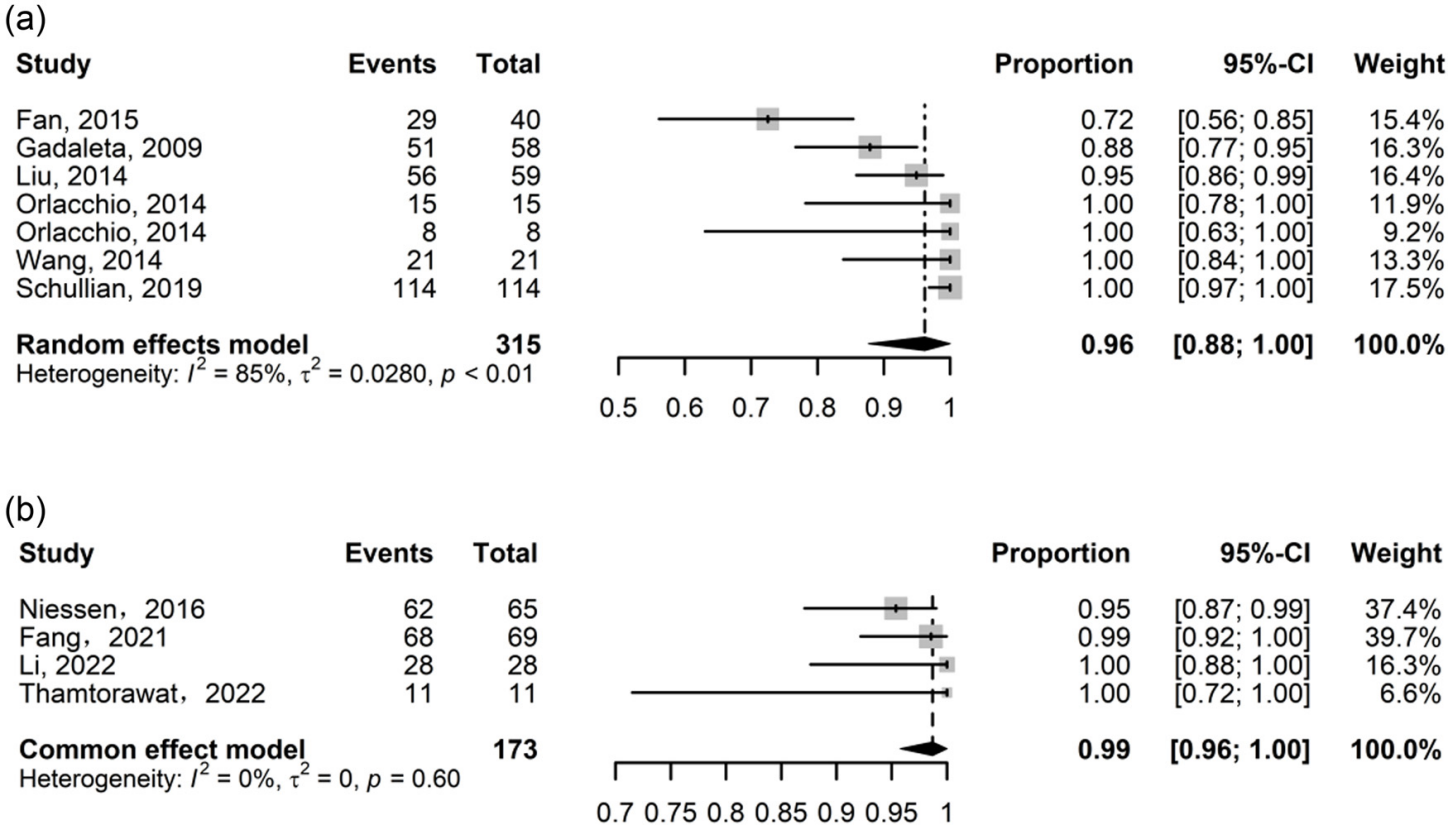
Forest plot for technical success rate for (a) RFA and (b) IRE.

Data on ablation success rates 1 month following RFA were published in 24 studies (*N* = 1,685), with results ranging from 63 to 97%. The pooled proportion using a random effect model (*I* ^2^ = 74%, *P* < 0.01) was 86% (95% CI: 82–89%) (Figure 3). In contrast, only 13 studies (*N* = 524) of IRE provided data on ablation success rates ranging from 72 to 100%. Also employing a random effects model (*I* ^2^ = 62%, *P* < 0.01), the result was 87% (95% CI: 81–92%) (Figure 2).

Fewer studies reported the technical success rate, with seven (*N* = 315) studies in RF and four (*N* = 173) in IRE. The pooled technique success rate of RFA using a random effect model (*I* ^2^ = 85%, *P* < 0.01) was 96% (95% CI: 88–100%). There was no heterogeneity in the IRE meta-analysis (*I* ^2^ = 0%, *P* = 0.60), so the pooled proportion of the fixed-effect model was 99% (95% CI: 96–100%) (Figure 3).

**Figure 4:**
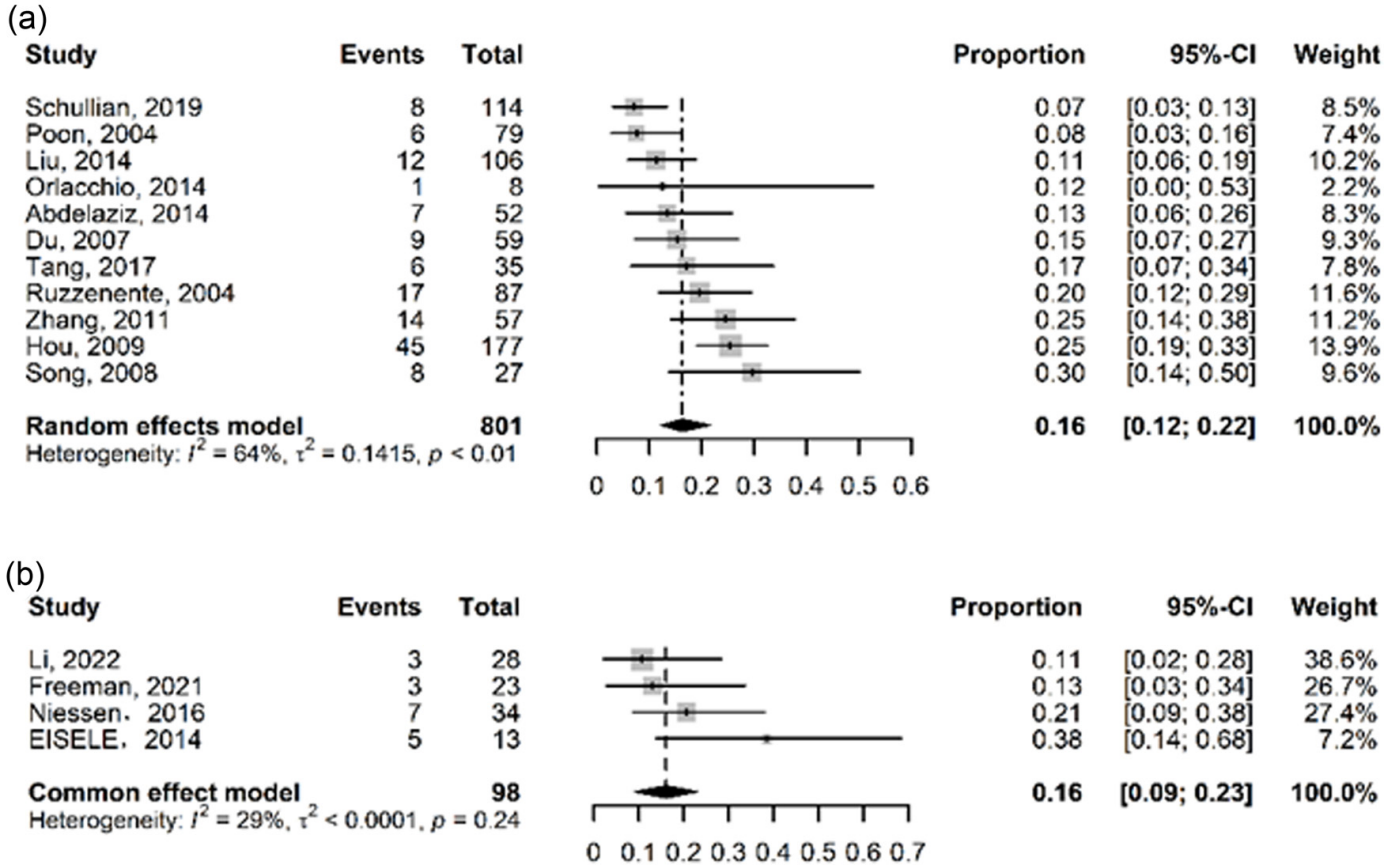
Forest plot for recurrence rate for (a) RFA and (b) IRE.

RFA recurrence rates were reported in 11 research, ranging from 7 to 30%, with heterogeneity among research (*I* ^2^ = 64%, *P* < 0.01), so random-effects models were used, resulting in 16% (95% CI: 12–22%). IRE recurrence rates were reported in four research, ranging from 11 to 38%, with no heterogeneity among studies (*I* ^2^ = 29%, *P* = 0.24) and a fixed-effects model result was 16% (95% CI: 9–23%) (Figure 4).

### 3.3 Assessment of safety outcomes

**Figure 5:**
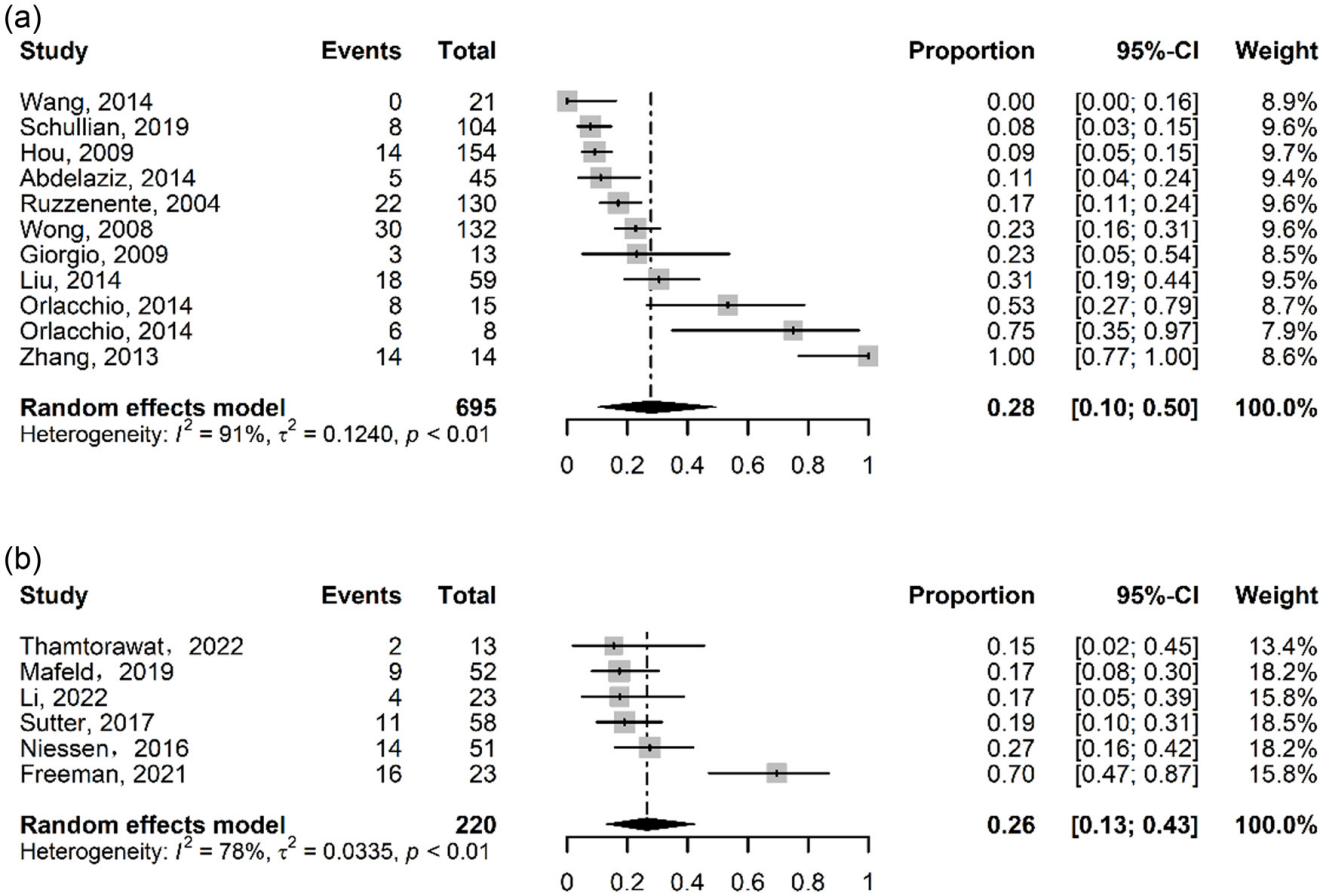
Forest plot for complications rate for (a) RFA and (b) IRE.

In this study, only the total complication rate was analyzed. There were 11 research (*N* = 695) that reported total complication rates for RAF. The complication rates reported by different research ranged significantly from 0 to 100%, and *I* ^2^ also similarly showed a large heterogeneity between research (*I* ^2^ = 91%, *P* < 0.01). As a result, the pooled incidence rate using a random effect mode was 28% (95% CI: 10–50%). There were six studies (*N* = 220) that reported total complication rates for IRE, ranging from 15 to 70% with high heterogeneity (*I* ^2^ = 78%, *P* < 0.01), and the result of the random-effects model was 26% (95% CI: 13–43%) (Figure 5).

### 3.4 Risk of bias

**Figure 6:**
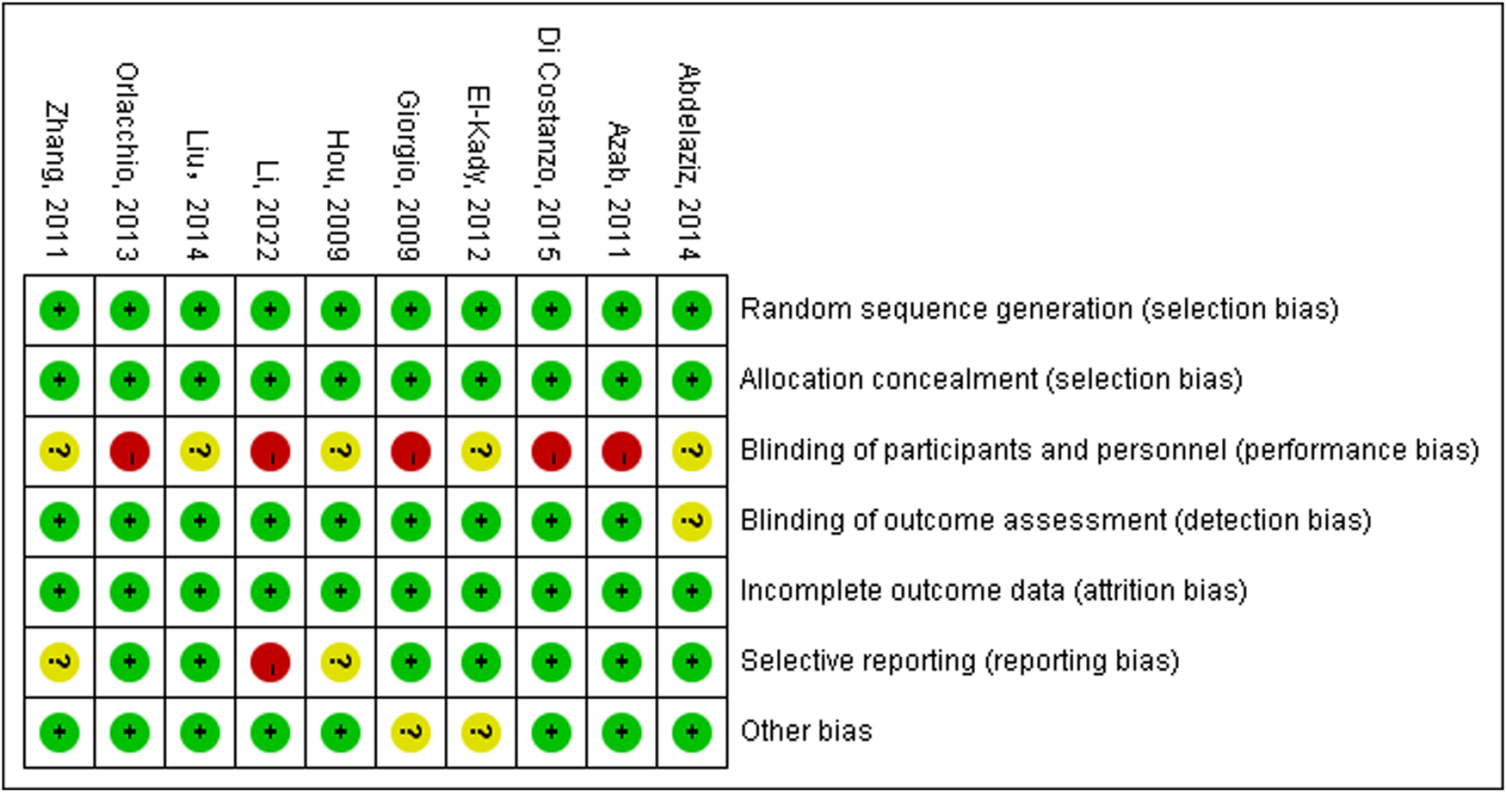
Cochrane ROB result of included studies.

Of the 37 articles, 27 cohort studies were assessed by NOS and found to be 6 and higher (Table 2), and the Cochrane ROB assessments of ten RCTs are shown in Figure 6. It is worth mentioning that since both RFA and IRE are surgical treatment methods, randomized controlled trials cannot meet the criteria for performance bias in Cochrane ROB.

**Table 2:** NOS of the included studies

Study	Year	Selection	Comparability	Outcome	Quality scores (maximum 9)
Liu	2014	**	*	***	6
Huang	2009	**	*	***	6
Song	2012	**	*	***	6
Fan	2015	**	*	***	6
Du	2007	**	*	***	6
Wang	2014	**	*	***	6
Song	2008	***	*	***	7
Zhang	2013	**	*	***	6
Tang	2017	***	*	***	7
Schullian	2019	***	*	***	7
Sutter	2017	***	*	***	7
Freeman	2021	**	*	***	6
Cheung	2013	**	*	***	6
Mafeld	2019	***	*	***	7
Niessen	2017	***	*	***	7
Beyer	2016	***	*	***	7
Fang	2021	***	*	***	7
Thamtorawat	2022	***	*	***	7
Cheng	2015	**	*	***	6
GADALETA	2009	***	*	***	7
Poon	2004	***	*	***	7
Orlacchio	2014	**	*	***	6
Ruzzenente	2004	***	*	***	7
Zhou	2020	**	*	***	6
EISELE	2014	**	*	***	6
Wong	2008	***	*	***	7
Niessen	2016	**	*	***	6

## 4 Discussion

The treatment of liver cancer is characterized by multidisciplinary involvement and the coexistence of multiple treatment methods. Common treatment methods include hepatectomy, liver transplantation, ablation therapy, TACE, radiation therapy, systematic anti-tumor therapy, etc. By selecting appropriate treatment modalities for patients at different stages of liver cancer, the curative effect can be maximized. Although surgery is considered the first choice for the radical treatment of liver cancer, many patients suffer from varying degrees of cirrhosis and some cannot tolerate surgical intervention. In recent years, image-guided local ablation has become increasingly significant and widely used in the treatment of liver cancer due to the rapid development of minimally invasive interventional therapy technology. At present, commonly utilized ablation procedures including MWA and RFA are notable for their precise efficacy, less trauma, and minimal influence on liver function. In some patients with early liver cancer, similar results can be obtained as surgical resection (SR).

RFA is considered a viable alternative to SR as it uses high-frequency electrical currents to heat tissue, leading to the coagulative necrosis of tumor cells. However, when the temperature reaches 100°C, tissue desiccation (charring) occurs, which leads to an increase in tissue impedance and affects energy transfer. At the same time, the heat around the electrode will be lost with the adjacent blood perfusion, i.e., the “heat-sink” effect, which may lead to incomplete ablation [42]. The effectiveness of RFA in achieving complete tumor eradication depends on tumor size and location. Tumors adjacent to vessels larger than 3 mm in diameter have a higher risk of ablation failure and local tumor progression [43]. In 2005, Lu et al. found the average maximum diameter of successfully treated lesions was 2.0 cm, while that of failure was 3.1 cm, and only 47% of perivascular lesions were successfully treated [44]. Other clinical investigations’ findings have demonstrated that lesions located under the gallbladder pericardium or near the gallbladder have an increased risk of incomplete ablation and local recurrence [45,46]. Therefore, RFA has certain limitations in focal ablation therapy.

In contrast, IRE avoids the thermal deposition effect by perforating cell membranes with an electric field, making it tissue-selective and avoiding thermal ablation. As a result, lesions around bile ducts and perivascular can be removed without affecting their function [47]. The IRE device achieved a comparable ablation effect on liver cancer to that of RFA. It should be noted that the ablation time of IRE was significantly prolonged, which may be related to the large number of discharge electrodes, unstable heart rates, and interference of ECG synchronization during ablation [47]. Theoretically, IRE can solve the problem of ablating lesions in special areas. In pig experiment, Rubinsky et al. discovered that IRE could rapidly ablate tissue next to big venous systems while maintaining the structural integrity of the portal vein and bile ducts [48]. Additionally, animal experiments conducted by Charpentier et al. have shown that IRE could safely ablate periportal tissues without causing collateral damage to the bile ducts, hepatic arteries, and portal vein [49]. Numerous clinical trials have shown that IRE can safely ablate liver tumors near critical structures where thermal ablation is contraindicated [50,51]. A study by Thomson et al. on the safety of IRE in humans found that the adverse effects of IRE on blood vessels near tumors were acceptable [52]. A systematic review of IRE in patients with liver cancer showed that the complete response rate at 3 months ranged from 67 to 100% for tumors bigger than 3 cm and from 93 to 100% for tumors less than 3 cm, and reported no major adverse events [53].

The effectiveness and safety of RFA and IRE have previously been compared in several articles in terms of tumor ablation success, complication, and tumor recurrence rate [40,54,55]. There has been no other meta-analysis discussing the ablation success rate 1 month after ablation. In contrast, the General Office of the National Health and Wellness Commission of China’s Guidelines for the Diagnosis and Treatment of Primary Liver Cancer (2022 Edition) [56] recommends reviewing dynamic enhanced CT, multiparametric MRI scanning, or ultrasonography about a month after ablation to assess the ablation effect. The European Association for the Study of the Liver recommends that localized treatments need to be carefully evaluated by CT at least 4 weeks after surgery [57]. Additionally, in many clinical studies, researchers determine whether patients require a second ablation and develop follow-up treatment plans based on the ablation results observed 1 month after the procedure [16,28,29,32]. Therefore, short-term ablation success data around 1 month are crucial for clinical treatment strategies. This review focuses on literature reporting this metric and verifies that the effectiveness and safety of IRE are comparable to RFA, providing valuable evidence for clinical assessment of efficacy, patient follow-up, and prognosis after IRE. These data also serve as a reference for designing future clinical trial protocols for liver cancer ablation, including assumptions of success rates and sample size calculations.

In addition to the ablation success rate, this article analyzes the technical success rate, recurrence rate, and complication for both techniques. The results of the analysis of the 37 included articles showed that the success rates of IRE and RFA ablation after about 1 month were 86% (95% CI: 82–89%) and 87% (95% CI: 81–92%), respectively. Technical success rates were 96% (95% CI: 88–100%) and 99% (95% CI: 96–100%). In addition, the recurrence rate of RFA group was 16% (95% CI: 12–22%) and IRE group was 16% (95% CI: 9–23%). In terms of safety, the RFA had a complication rate of 28% (95% CI: 10–50%) and the IRE had a rate of 26% (95% CI: 13–43%). Radiofrequency has been on the market for more than 20 years now, both product technology and the skill of operating physicians are becoming more mature. As a newer technology, IRE products are continuously being improved, and the proficiency of physicians in performing the procedure is also increasing. This is reflected in the trend of rising ablation success rates for IRE, which have increased from 72% in previous years to 100% in 2020.

In conclusion, the results of these studies selected in this meta-analysis showed that IRE ablation for liver cancer is safe and effective, and its ablation success rate and complication rate are similar to those of RFA. We anticipate that, with ongoing clinical practice and application, this technique will continue to reduce complication rates and achieve even better outcomes.

## 5 Conclusion

In patients with liver cancer receiving focal therapy, the ablation success rate of IRE was similar to RFA at about 1 month after surgery. IRE can also ablate some special sites, which has a broad development prospect in the treatment of solid tumors.
